# Discovery and ranking of the most robust prognostic biomarkers in serous ovarian cancer

**DOI:** 10.1007/s11357-023-00742-4

**Published:** 2023-03-01

**Authors:** Balázs Győrffy

**Affiliations:** Dept. of Bioinformatics, Semmelweis University, Tuzolto U. 7-9, 1094 Budapest, Hungary

**Keywords:** Gene expression, Gene arrays, Survival, Cox regression

## Abstract

Progress in ovarian cancer treatment lags behind other tumor types. With diagnosis usually at an advanced stage, there is a high demand for reliable prognostic biomarkers capable of the selection of effective chemo- and targeted therapies. Our goal was to establish a large-scale transcriptomic database and use it to uncover and rank survival-associated genes. Ovarian cancer cohorts with transcriptome-level gene expression data and clinical follow-up were identified from public repositories. All samples were normalized and entered into an integrated database. Cox univariate survival analysis was performed for all genes and was followed by multivariate analysis for selected genes involving clinical and pathological variables. False discovery rate was computed for multiple hypothesis testing and a 1% cutoff was used to determine statistical significance. The complete integrated database comprises 1816 samples from 17 datasets. Altogether, 2468 genes were correlated to progression-free survival (PFS), and 704 genes were correlated with overall survival (OS). The most significant genes were WBP1L, ASAP3, CNNM2, and NCAPH2 for progression-free survival and CSE1L, NUAK1, ALPK2, and SHKBP1 for overall survival. Genes significant for PFS were also preferentially significant for predicting OS as well. All data including HR and *p* values as well as the used cutoff values for all genes for both PFS and OS are provided to enable the ranking of future biomarker candidates across all genes. Our results help to prioritize genes and to neglect those which are most likely to fail in studies aiming to establish new clinically useful biomarkers and therapeutic targets in serous ovarian cancer.

**Supplementary Information:**

The online version contains supplementary material available at 10.1007/s11357-023-00742-4.

## Introduction

With a 64.4% mortality rate, epithelial ovarian cancer remains the most lethal malignancy of the female genital system [1]. Surgery represents the most effective treatment, but without an established screening test, the diagnosis is frequently in advanced stages where surgical removal is not possible anymore. Ovarian cancer has a significant genetic component, and population-based screenings show that 14.5% of all patients have at least one pathogenic variant with BRCA1, BRCA2, CHEK2, BRIP1, and MSH2 being the most prevalent ones [2]. The major histological subtypes of epithelial ovarian cancer include the serous, endometrioid, clear cell, and mucinous subtypes [3]. Of these, the serous subtype, which can be broken down into high-grade serous tumors and low-grade serous tumors, accounts for over four quarters of all cases [4].

Chemotherapy is the only treatment option in a high proportion of patients with advanced disease. The most commonly used systemic therapy agents comprise carboplatin and paclitaxel [5]. Other commonly used chemotherapy drugs include cisplatin, docetaxel, and liposomal doxorubicin [6]. About 15% of ovarian cancer patients have a germline mutation in the BRCA1 or BRCA2 genes, and in these cases, chemotherapy can be supplemented with the administration of PARP inhibitors, which limit the regeneration of the cells as well as the further growth and proliferation of the tumor cells [7].

In oncology, gene expression–based, mutation-based, and protein abundance–based biomarkers can be employed for screening and treatment selection. In ovarian cancer, CA125 levels were previously determined for screening. However, CA125 is not recommended anymore, as it was unable to reach sufficiently strong sensitivity and specificity necessary for a reliable clinical application in population-wide screenings [6]. Following the removal of the primary source of the tumor, the median progression-free survival in ovarian cancer is 18 months, and rising CA125 levels can be used as a biomarker for the early detection of relapse [3]. Despite improvements in combination chemotherapy, systemic resistance and consequent relapse are major factors limiting the expected survival [8]. To date, there is no established biomarker capable to predict response to systemic chemotherapy. In this field, numerous mRNA-, protein-, and DNA-based biomarker candidates have been proposed to improve patient selection for specific treatments, but none of them was approved for clinical application. A few representative examples include CHI3L1 [9] for paclitaxel resistance and CSF1R [10], TRO [11], and OXCT1 [12] for cisplatin resistance. A comprehensive review of predictive biomarker candidates linked to chemotherapy resistance was published previously [8].

Another group of biomarkers comprise prognostic biomarkers, which are able to identify the likelihood of a future clinical event like recurrence or death from the disease or after a specific medical treatment. Prognostic biomarkers can be employed to select more aggressive or combination therapies. Almost two hundred prognostic biomarker candidates for ovarian cancer have been reviewed recently [13]. Nevertheless, new candidates were also published last year including single genes [14, 15] and gene signatures [16].

In this study, our goal was to uncover and rank gene expression–based biomarkers correlated to prognosis in serous ovarian cancer. In particular, we had the following three objectives: first, we aimed to establish an integrated large-scale transcriptomic database of ovarian cancer cases with pathological and follow-up data which can be utilized for biomarker discovery. Secondly, we utilized this cohort to uncover genes with the highest correlation to survival in serous ovarian cancer. Third, we aimed to assess the capability of progression-free survival–associated biomarkers to predict overall survival. Overall, our results help to prioritize genes in studies aiming to uncover new clinically useful biomarkers and therapeutic targets.

## Methods

### Database setup

We have searched for ovarian cancer cohorts in NCBI Gene Expression Omnibus (https://www.ncbi.nlm.nih.gov/geo/) and in the Genomic Data Commons Data Portal (https://portal.gdc.cancer.gov/). Only samples with available transcriptome-level data with a minimum of ten patients were considered. To avoid differences due to different sensitivity, specificity, and dynamic range in detecting gene expression levels for specific genes by different technologies, we narrowed the search only to tumor samples examined using the in situ oligonucleotide array platforms GPL96 (Affymetrix Human Genome U133A Array), GPL571 (Affymetrix Human Genome U133A 2.0 Array), and GPL570 (Affymetrix Human Genome U133 Plus 2.0 Array). The advantage of these arrays is that they use identical probe sequences to measure the expression of individual genes.

### Data processing

The oligonucleotide gene array files were MAS5 normalized. Then, a second scaling normalization was executed to set the mean expression in each array to 1000. Only the probes present in the GPL96 platform were used in the scaling normalization to prevent platform-specific differences due to the higher probe number in the GPL570 arrays. Quality control was executed by checking the background intensity, the noise, the percentage of present calls, the presence of bioBCD spikes, and the GAPDH/ACTB 3 to 5 ratio. JetSet was used to select the most reliable probe set for each gene [17]. Finally, cellular content for immune infiltration was determined using xCell [18].

Clinical data were extracted from the supplemental material of the original publications or from the series matrix files in GEO. For each sample, overall survival (OS) time and event, progression-free survival (PFS) time and event, histological subtype, stage, grade, the success of debulking, TP53 mutation status, and treatment data were recorded whenever available. Debulking surgery, sometimes called ovarian cytoreduction surgery, is performed after the elimination of the ovaries and includes the removal of cancerous tumors from the pelvis. Generally, debulking surgery cannot lead to complete elimination of all tumor tissue, and the remaining cells can form a residual disease. For this reason, progression-free survival is published usually in ovarian cancer, which refers to time from treatment (surgery) to further disease progression in the patients.

### Statistical analysis

Cox proportional hazards regression was used to compute differential survival. First, a univariate analysis was performed for each gene separately. To avoid missing correlations due to the use of a specific cutoff, all available cutoff values between the lower and upper quartiles of expression were used for each gene, and false discovery rate (FDR) using the Benjamini–Hochberg method was computed to correct for multiple hypothesis testing [19]. The cutoff value with the highest significance (lowest FDR) was determined — in case of multiple cutoff values with identical significance, the cutoff with the highest hazard (HR) rate was selected for the final analysis. When determining the top genes with the most reliable correlation to survival, only genes with a cutoff value over 200 were considered — this is twice the background intensity of about 100. In addition, preference was given to genes linked to worse prognosis with higher expression, as these genes represent optimal future therapeutic targets. For this selected set of top genes, multivariate Cox regression was computed to evaluate the effect on survival of clinical and pathological variables and gene expression. Kaplan–Meier plots were drawn to visualize survival differences using the cutoff values determined in the univariate analysis.

### Analysis platform extension

Our previously established Kaplan–Meier plotter (https://www.kmplot.com) was extended to include the complete integrated ovarian cancer database. The platform can be used to validate the findings of the present study in real time as well as for the future corroboration of new gene expression–based biomarker candidates and gene signatures in the present and additional sub-cohorts of patients not investigated in the current study.

### Gene ontology analysis

To uncover higher-level functions related to altered progression-free survival, gene ontology analysis was performed using the enrichGO function in the TNM plotter (http://www.tnmplot.com) [20]. Two separate analyses were performed. The first analysis used all significant genes with an FDR below 0.01, where higher expression correlates to worse survival, and a second one for genes, where higher expression correlates to improved survival. Both analyses were performed to uncover significant biological processes.

## Results

### Integrated database

**Fig. 1:**
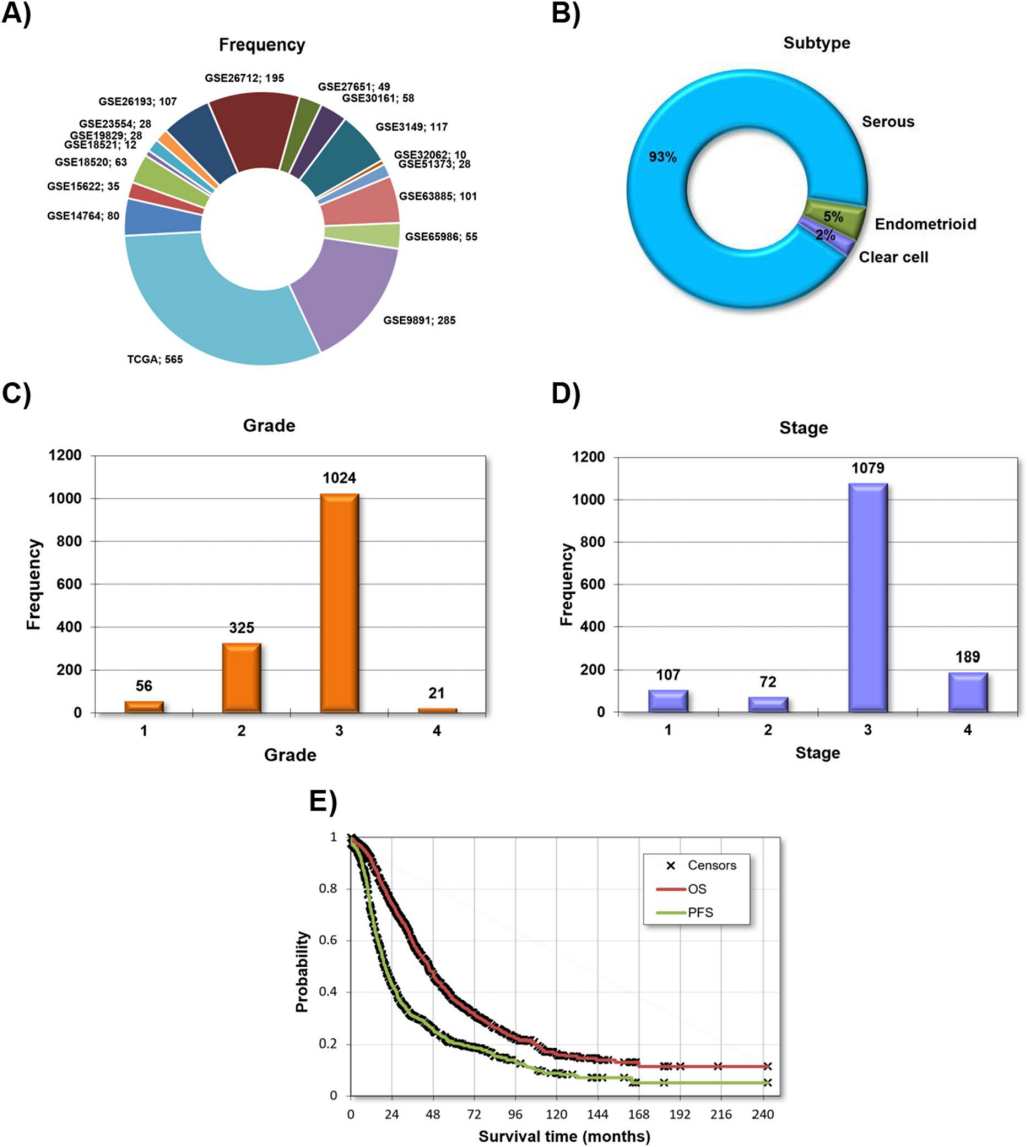
Clinical and pathological characteristics of all samples included in the entire database including a list of all datasets (**A**), distribution of the three histological subtypes (**B**), grade (**C**), stage (**D**), and a Kaplan–Meier plot showing both overall survival (OS) and progression-free survival (PFS) for all patients (**E**)

The complete database includes all together 1816 samples from 17 independent datasets. The largest cohorts are from the TCGA and GSE9891 studies, which provide almost half of all the patients. Notably, none of the clinical parameters was available in each dataset. About 93% of all samples belong to the serous subtype, and most of the subsequent analyses were restricted to this cohort. Most of the patients were diagnosed with stage 3 and grade 3 diseases. Across all patients with available data, debulking was successful in 59.9% of the patients. The average follow-up was 24.9 months for progression-free survival and 39.1 months for overall survival. The detailed clinical and pathological characteristics for each dataset are summarized in Table 1 and available treatment agents in Supplemental Table 1, and the most important features are depicted in Fig. 1.

**Table 1:** Summary of cohorts included in the integrated database

Cohort	*n*	%	Survival				Histology			Stage				Grade				TP53 mutation		Debulk	
			OS *n*	OS follow-up (months)	PFS *n*	PFS follow-up (months)	Serous	Endometrioid	Clear cell	I	II	III	IV	1	2	3	4	Wild type	Mutated	Suboptimal	Optimal
GSE14764	80	4.41	80	33.7 ± 15.3	80	21.23 ± 14.7	90.7% (68)	9.3% (7)	0%	10.0% (8)	1.3% (1)	86.3% (69)	2.5% (2)	3.8% (3)	28.8% (23)	67.5% (54)	0% (0)	----	----	6.9% (2)	93.1% (27)
GSE15622	35	1.93	35	37.9 ± 27.6	35	25.0 ± 24.1	100 % (31)	0%	0%	0% (0)	0% (0)	74.3% (26)	25.7% (9)	0% (0)	20.0% (7)	80.0% (28)	0% (0)	----	----	----	----
GSE18520	63	3.47	53	40.4 ± 41.3	----	----	100% (53)	0%	0%	----	----	----	----	0% (0)	0% (0)	100.0% (53)	0% (0)	----	----	----	----
GSE18521	12	0.66	----	----	----	----	----	----	----	----	----	----	----	----	----	----	----	----	----	----	----
GSE19829	28	1.54	28	43.0 ± 30.4	----	----	----	----	----	----	----	----	----	----	----	----	----	----	----	----	----
GSE23554	28	1.54	28	49.3 ± 45.4	----	----	100% (28)	0%	0%	----	----	----	----	7.1% (2)	28.6% (8)	64.3% (18)	0% (0)	----	----	51.8% (14)	48.2% (13)
GSE26193	107	5.89	107	49.9 ± 39.6	107	36.4 ± 39.2	84.9% (79)	8.6% (8)	6.5% (6)	18.7% (20)	10.3% (11)	55.1% (59)	15.9% (17)	6.5% (7)	30.8% (33)	62.6% (67)	0% (0)	16.2% (11)	83.8% (57)	64.5% (69)	35.5% (38)
GSE26712	195	10.74	185	47.6 ± 36.5	185	47.6 ± 36.5	----	----	----	----	----	----	----	----	----	----	----	----	----	51.4% (95)	48.6% (90)
GSE27651	49	2.70	39	57.5 ± 49.0	----	----	----	----	----	20.9% (9)	4.7% (2)	67.4% (29)	7.0% (3)	30.2% (13)	2.3% (1)	48.8% (21)	0% (0)	----	----	----	----
GSE30161	58	3.19	58	45.8 ± 35.6	54	24.7 ± 31.9	97.9% (47)	2.1% (1)	0%	0% (0)	0% (0)	91.4% (53)	8.6% (5)	3.7% (2)	35.2% (19)	61.1% (33)	0% (0)	----	----	53.6% (30)	46.4% (26)
GSE3149	117	6.44	117	49.0 ± 41.7	----	----	----	----	----	0% (0)	0.9% (1)	82.8% (96)	16.4% (19)	3.5% (4)	48.2% (55)	47.4% (54)	0.9% (1)	----	----	45.3% (53)	54.7% (64)
GSE32062	10	0.55	10	70.3 ± 42.7	10	16.8 ± 10.8	100% (10)	0%	0%	0% (0)	0% (0)	60.0% (6)	40.0% (4)	0% (0)	30.0% (3)	70.0% (7)	0% (0)	----	----	60% (6)	40% (4)
GSE51373	28	1.54	----	----	28	16 ± 11.5	----	----	----	0% (0)	18.5% (5)	70.4% (19)	11.1% (3)	----	----	----	----	----	----	----	----
GSE63885	101	5.56	75	42.8 ± 28.9	75	13.9 ± 20.9	85.9% (73)	14.1% (12)	0%	0% (0)	2.6% (2)	83.1% (64)	14.3% (11)	0% (0)	12.5% (10)	63.8% (51)	23.8% (19)	16.7% (15)	83.3% (75)	80% (60)	20% (15)
GSE65986	55	3.03	----	----	55	25.0 ± 20.1	29.1% (16)	25.5% (14)	45.4% (25)	54.5% (30)	9.1% (5)	20.0% (11)	16.4% (9)	----	----	----	----	----	----	----	----
GSE9891	285	15.69	285	31.1 ± 23.2	285	19.9 ± 17.8	93.0% (264)	7.0% (20)	0%	8.5% (24)	6.4% (18)	77.2% (217)	7.8% (22)	6.8% (19)	34.6% (97)	58.6% (164)	0% (0)	----	----	30.4% (70)	69.6% (160)
TCGA	565	31.11	557	33.3 ± 26.8	522	20.0 ± 19.9	100% (563)	0%	0%	2.9% (16)	4.8% (27)	77.1% (430)	15.2% (85)	1.1% (6)	12.5% (69)	85.9% (474)	0.2% (1)	16.5% (76)	83.4% (384)	27.3% (137)	72.7% (365)
Entire database	**1,816**		**1,657**	**39.1 ± 32.3**	**1,436**	**24.9 ± 26.3**	**93% (1,232)**	**4.7% (62)**	**2.3% (31)**	**7.4% (107)**	**5.0% (72)**	**74.5% (1,079)**	**13.1% (189)**	**3.9% (56)**	**22.7% (325)**	**71.8% (1,024)**	**14.1% (21)**	**16.5% (102)**	**83.5% (516)**	**40.1% (536)**	**59.9% (802)**

### Univariate analysis across all genes

**Fig. 2:**
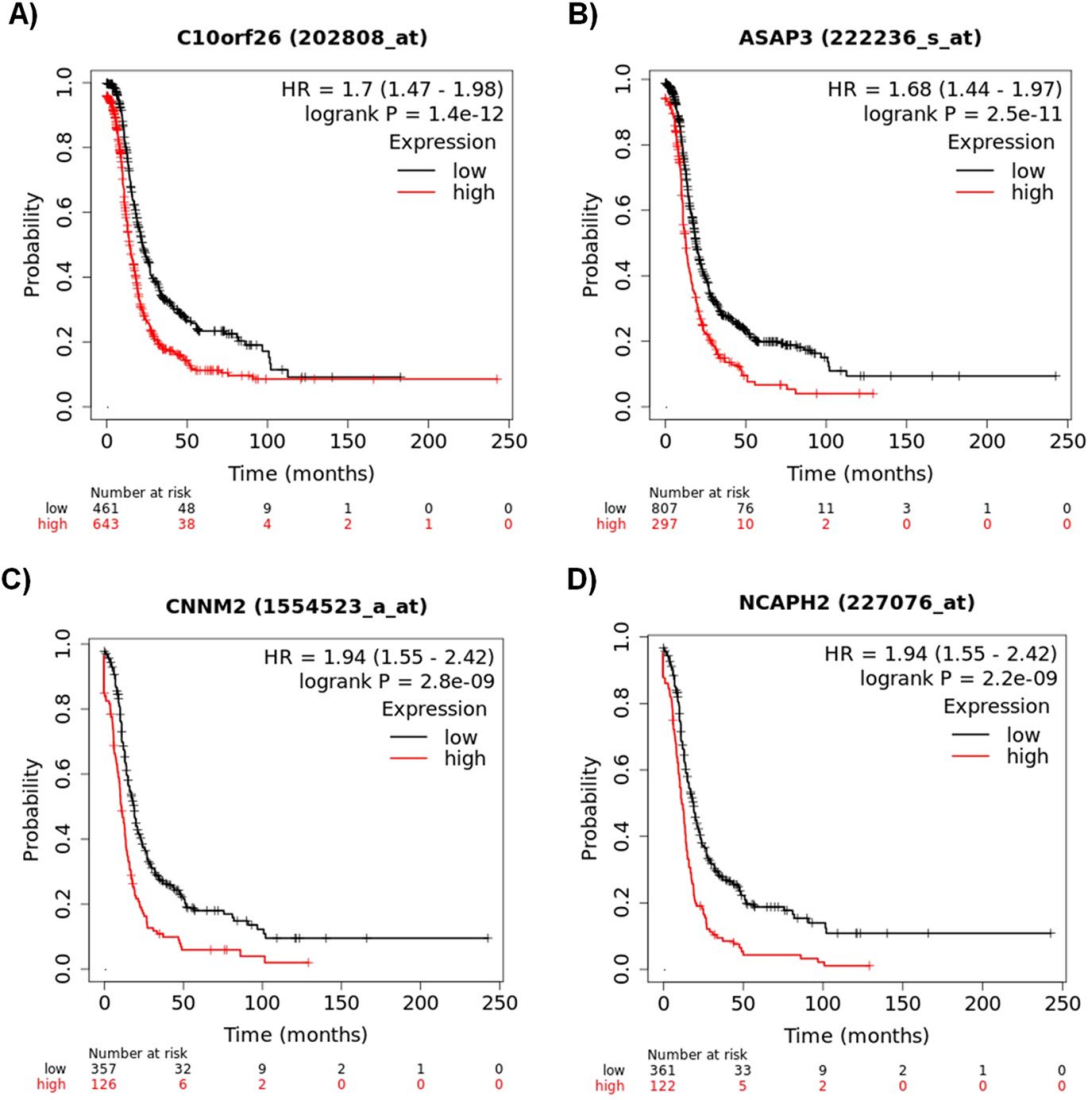
Most robust genes associated with PFS including C10orf26 (**A**) and ASAP3 (**B**) were measured by both GP96 and GPL570 arrays, and CNNM2 (**C**) and NCAPH2 (**D**) measured by GPL570 arrays only

First, univariate analysis was performed for all genes in all serous samples. When looking across all available genes related to progression-free survival in all patients using GPL96 probes (this computation is based on data from 1104 serous patients including both GPL96 and GPL570 samples with available PFS data), altogether, 1231 genes were significant. C10orf26 (also known as WBP1L, *p* = 1.4e − 12, HR = 1.7) and ASAP3 (*p* = 2.5e − 11, HR = 1.68) reached the highest significance (see Fig. 2 A and B for WBP1L and ASAP3, respectively). There were 483 patients with PFS data measured with the GPL570 platform only, and 1237 genes reached statistical significance. In this cohort, the two most significant genes were CNNM2 (*p* = 2.8e − 09, HR = 1.94, Fig. 2C) and NCAPH2 (*p* = 2.2e − 09, HR = 1.94, Fig. 2D).

**Fig. 3:**
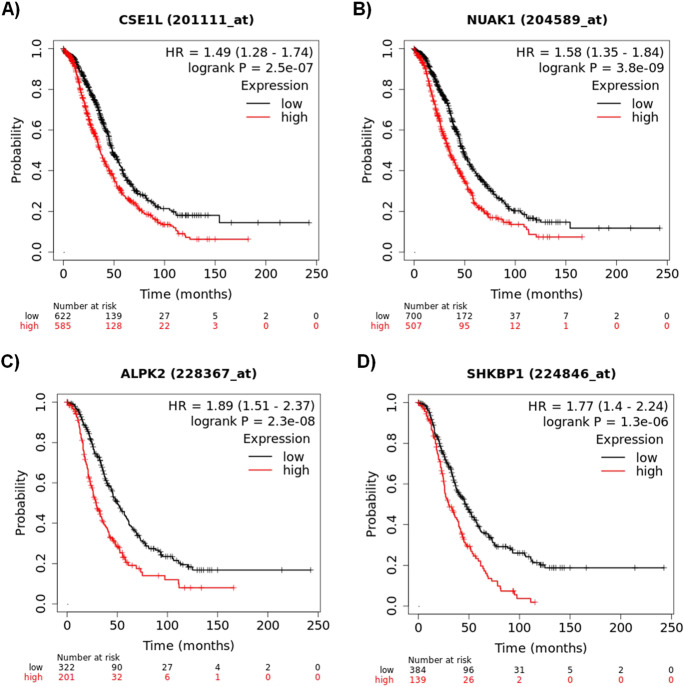
Most robust genes associated with OS including CSE1L (**A**) and NUAK1 (**B**) measured by both GPL96 and GPL570 arrays and ALPK2 (**C**) and SHKBP1 (**D**) were determined by GPL570 arrays only

When looking for markers linked with overall survival, the combined GPL96 + GPL570 cohort comprises 1207 serous patients with survival data. In this analysis, out of 340 significant genes, the most significant ones were CSE1L (*p* = 2.5e − 07, HR = 1.49, Fig. 3A) and NUAK1 (*p* = 3.8e − 09, HR = 1.58, Fig. 3B). The GPL570 cohort embraces 523 tumor samples with OS data. In this group, 364 genes reached an FDR below 1%, and the two most significant genes were ALPK2 (*p* = 2.3e − 08, HR = 1.89, Fig. 3C) and SHKBP1 (*p* = 1.3e − 06, HR = 1.77, Fig. 3D).

For PFS, the HR and *p* values as well as the used cutoff values for all genes in both platforms are listed in Supplemental Table 2. For OS, the analysis results for all genes measured in all platforms are provided in Supplemental Table 3. These tables enable the ranking of future biomarker candidates across all genes in serous ovarian cancer.

### Multivariate analysis

The top four genes with the highest prognostic power for progression-free survival were further analyzed in a multivariate analysis to assess their independence from established pathological parameters. When compared with stage, grade, and debulking success, all four genes including WBP1L (*p* = 2.2e − 06), ASAP3 (4.9e − 06), CNNM2 (6.8e − 04), and NCAPH2 (*p* = 8.3e − 04) retained high significance. At the same time, stage, grade, and debulking success were also significant suggesting that the different genes and clinicopathological features capture independent, prognostically relevant features of the tumor.

### Tumor-infiltrating lymphocytes

Cellular infiltration was determined using the gene expression signature across all genes in xCell for B-cells, CD4 + T-cells, CD4 + memory T-cells, CD8 + T-cells, CD8 + naive T-cells, dendritic cells, eosinophils, macrophages, monocytes, NK cells, neutrophils, and regulatory T-cells. Of these, higher presence of B-cells (*p* = 1.6e − 05) and monocytes (*p* = 1.2e − 06) was linked to shorter PFS, while higher presence of dendritic cells was linked to longer PFS (*p* = 1.7e − 06). However, when running multivariate analysis with WBP1L, ASAP3, CNNM2, and NCAPH2, only dendritic cells retained significance while each of the four genes was significant.

### Progression-free survival vs overall survival

To compare genes related to progression-free survival to those linked to overall survival, we performed a simplified analysis: the correlation was restricted to include all samples and all genes measured with the GPL96 platform and reaching a significant correlation with survival with a *p*-value below 0.01. In this setting, 6633 genes were not significant, 1824 genes were significant for PFS only, and 814 genes were significant for OS only. The overlap between the genes significant with both OS and PFS includes 819 genes. The number of genes significant is approximately twice the number of genes expected based on the observed proportions and reaches a *p* < 1e − 05 in a chi-square statistic.

### Gene ontology analysis results

The list of genes related to PFS with an FDR below 1% with a cutoff value over 200 and higher expression was selected for gene ontology analysis. The complete gene list including the combined GPL96 and GPL570 gene sets consists of 792 genes. The most significant biological processes were extracellular matrix organization (*p* = 3.2e − 08) and cellular response to TGF beta stimulus (*p* = 6.7e − 04). The complete list of all affected biological processes is provided in Supplemental Table 4. The downregulated gene list in this cohort includes 180 genes, and the most significant biological processes related to these features include translational initiation (*p* = 1.8e − 06) and nuclear-transcribed mRNA catabolic process (*p* = 1.9e − 05). The entire list of significant biological processes correlated to the downregulated list is available in Supplemental Table 5.

## Discussion

As current therapies for ovarian cancer are far from optimal, there is an imperative need to identify new therapeutic targets. Here, we have set up a framework for the transcriptome-based discovery of genes significantly related to ovarian cancer prognosis. The established ranking can help to compare targets of systemic therapies available in ovarian cancer, including chemotherapy and established targeted therapy options [21], as well as the targets of new agents currently in clinical development [22]. Notably, our approach can also be applied to genes uncovered in preclinical models. For example, non-mutated genes highly expressed in ovarian cancer were tested in cell lines via RNAi to identify such new targets [23].

A recent development is the broadening use of immunotherapy in the systemic therapy of various solid tumors. However, ovarian cancer lags behind as minimal benefit with only few complete responses could be observed in trials investigating ovarian cancer patients with avelumab [24] and pembrolizumab [25] therapies. Previously, fourteen immune genes were suggested as potential prognostic biomarkers in ovarian cancer [26]. Our analysis also includes the fourteen genes, of which MAL, SCRN1, MIF, and KIFAP3 were significant in our combined cohort. Thus, here, we provide independent validation for the prognostic biomarker potential of these genes.

Instead of OS, PFS is frequently used as a surrogate endpoint in basic studies and in clinical trials for ovarian cancer [27]. However, this approach is frequently debated [28], and in our case, a fundamental question arises as of what proportion of genes related to PFS is also linked to OS? To respond to this question, we determined the proportion of genes linked to PFS and OS simultaneously. In this analysis, the measured number of genes linked to both survival settings was almost twice the expected proportion and delivered high significance. In other words, these results prove that genes significant for PFS are preferentially significant for OS as well.

Our results have uncovered the genes most significantly correlated to progression-free survival. Interestingly, none of the four top genes WBP1L, ASAP3, CNNM2, and NCAPH2 was correlated with ovarian cancer pathogenesis previously. WBP1L (WW domain binding protein 1 Like) is a regulator of CXCR4 signaling and hematopoiesis [29]. ASAP3 (ArfGAP With SH3 domain, ankyrin repeat and PH domain 3) promotes cell differentiation and migration and has been previously linked to cancer cell invasion [30]. CNNM2 (cyclin and CBS domain divalent metal cation transport mediator 2) mediates the epithelial transport and renal reabsorption of Mg2 + [31]. NCAPH2 (non-SMC condensin II complex subunit H2) has a role in mitotic chromosome assembly [32]. We have to note that genes associated with prognosis are not necessarily target genes or druggable by currently available therapies. Further investigation using in vitro model systems, pharmacologic inhibition, and validation of these genes in independent cohorts of clinical patients will be needed to establish their biological properties and potential utility in ovarian cancer.

We have to note some limitations of our study. First, we had to restrict the analysis to serous tumors. Higher sample numbers would enable a reliable analysis for other subtypes of ovarian cancer like endometrioid or clear cell tumors. Second, the analysis only includes gene expression values. A protein-level analysis would not only enable the direct validation of gene function but would also enable an immunohistochemistry-based test. Nevertheless, we might overcome this limitation in the future as we could previously perform protein-level analysis using breast cancer samples [33]. Third, the clinical and pathological data were not available for all samples, and this prevented performing a multivariate analysis using all patients.

In summary, we established an integrated large-scale transcriptomic database of epithelial ovarian cancer cases with pathological and follow-up data which can be utilized for biomarker discovery. We then utilized this cohort to uncover the top genes with the highest correlation to progression-free and overall survival in serous ovarian cancer patients. Our results help to prioritize genes in studies aiming to identify new clinically useful biomarkers and therapeutic targets in serous ovarian cancer.

## Supplementary Information

Below is the link to the electronic supplementary material.

Supplementary file1 (DOCX 34 KB)

Supplementary file2 (XLSX 933 KB)

Supplementary file3 (XLSX 916 KB)

Supplementary file4 (XLSX 42.1 KB)

Supplementary file5 (XLSX 17.1 KB)
