# Supplementary material for: Discovery and ranking of the most robust prognostic biomarkers in serous ovarian cancer
Source: GeroScience. 2023 Mar 1;45(3):1889–98. doi: 10.1007/s11357-023-00742-4 (PMC10400493; doi:10.1007/s11357-023-00742-4)
Supplement: Supplementary file 1 — Supplementary file1 (DOCX 34 KB) [file 11357_2023_742_MOESM1_ESM.docx]

# Supplemental Table 1. Summary of the therapies administered to the patients in the different datasets.

| **Therapy includes** | | **Platinum** | **Taxol** | **Avastin** | **Docetaxel** | **Gemcitabine** | **Paclitaxel** | **Topotecan** |
| --- | --- | --- | --- | --- | --- | --- | --- | --- |
| **GSE14764** | no | 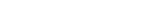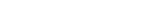1 | 0 | 0 | 0 | 0 | 0 | 0 |
|  | yes | 78 | 79 | 0 | 0 | 0 | 0 | 0 |
| **GSE15622** | no | 15 | 20 | 0 | 0 | 0 | 0 | 0 |
|  | yes | 20 | 15 | 0 | 0 | 0 | 0 | 0 |
| **GSE18520** | no | 0 | 0 | 0 | 0 | 0 | 0 | 0 |
|  | yes | 0 | 0 | 0 | 0 | 0 | 0 | 0 |
| **GSE18521** | no | 0 | 0 | 0 | 0 | 0 | 0 | 0 |
|  | yes | 0 | 0 | 0 | 0 | 0 | 0 | 0 |
| **GSE19829** | no | 0 | 0 | 0 | 0 | 0 | 0 | 0 |
|  | yes | 0 | 0 | 0 | 0 | 0 | 0 | 0 |
| **GSE23554** | no | 0 | 0 | 0 | 0 | 0 | 0 | 0 |
|  | yes | 28 | 0 | 0 | 0 | 0 | 0 | 0 |
| **GSE26193** | no | 0 | 0 | 0 | 0 | 0 | 0 | 0 |
|  | yes | 93 | 57 | 0 | 0 | 0 | 0 | 0 |
| **GSE26712** | no | 0 | 0 | 0 | 0 | 0 | 0 | 0 |
|  | yes | 185 | 0 | 0 | 0 | 0 | 0 | 0 |
| **GSE27651** | no | 0 | 0 | 0 | 0 | 0 | 0 | 0 |
|  | yes | 0 | 0 | 0 | 0 | 0 | 0 | 0 |
| **GSE30161** | no | 0 | 0 | 0 | 0 | 0 | 0 | 0 |
|  | yes | 58 | 54 | 0 | 0 | 0 | 0 | 0 |
| **GSE3149** | no | 0 | 22 | 0 | 0 | 0 | 0 | 0 |
|  | yes | 116 | 94 | 0 | 0 | 0 | 0 | 0 |
| **GSE32062** | no | 0 | 0 | 0 | 0 | 0 | 0 | 0 |
|  | yes | 10 | 10 | 0 | 0 | 0 | 0 | 0 |
| **GSE51373** | no | 0 | 0 | 0 | 0 | 0 | 0 | 0 |
|  | yes | 28 | 28 | 0 | 0 | 0 | 28 | 0 |
| **GSE63885** | no | 0 | 34 | 0 | 0 | 0 | 0 | 0 |
|  | yes | 75 | 41 | 0 | 0 | 0 | 0 | 0 |
| **GSE65986** | no | 0 | 0 | 0 | 0 | 0 | 0 | 0 |
|  | yes | 0 | 0 | 0 | 0 | 0 | 0 | 0 |
| **GSE9891** | no | 40 | 87 | 0 | 0 | 0 | 0 | 0 |
|  | yes | 242 | 195 | 0 | 0 | 0 | 0 | 0 |
| **TCGA** | no | 58 | 315 | 511 | 451 | 425 | 338 | 440 |
|  | yes | 505 | 248 | 50 | 108 | 135 | 220 | 119 |
